# Supplementary material for: Improved Medicago sativa Nodulation under Stress Assisted by Variovorax sp. Endophytes
Source: Plants (Basel). 2022 Apr 17;11(8):1091. doi: 10.3390/plants11081091 (PMC9026315; doi:10.3390/plants11081091)
Supplement: Supplementary file 1 [file plants-11-01091-s001.zip › plants-1661664-supplementary.pdf]

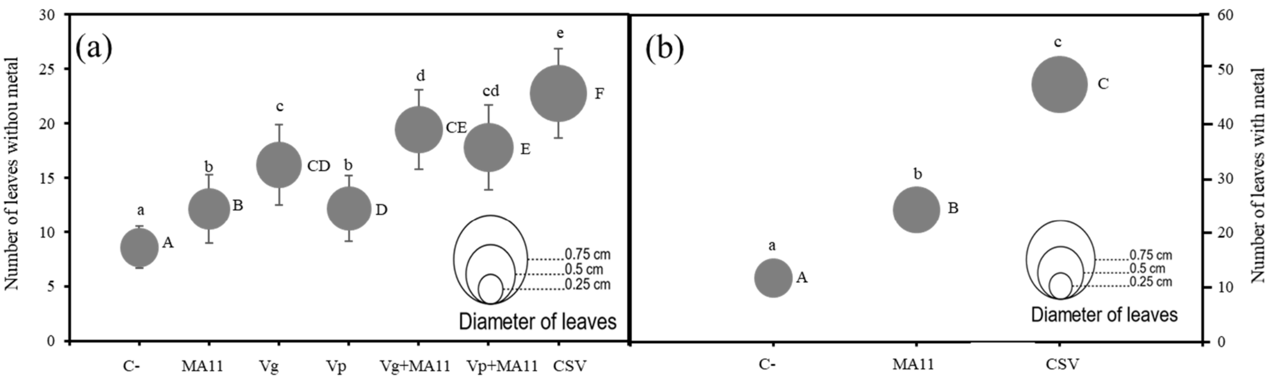

Figure S1. Number and size of *Medicago sativa* leaves in pot experiments. (**a**) Pots with nutrient-poor soil as a substrate and (**b**) pots with soils polluted with metal as a substrate after 60 days under greenhouse conditions. Values are means ± S.D. (n = 16). The size of a circle indicates the mean of the diameter of the leaves. Different letters indicate means that are significantly different from each other (one-way ANOVA; LSD test, $p < 0.0001$). C-: non inoculation; MA11: inoculation with *E. medicae* MA11; Vg: inoculation with *V. gossypii* JM-310^{T}; Vp: inoculation with *V. paradoxus* S110^{T}; Vg+MA11: co-inoculation with *V. gossypii* JM-310^{T} and *E. medicae* MA11; Vp+MA11: co-inoculation with *V. paradoxus* S110^{T} and *E. medicae* MA11; CSV: co-inoculation with *V. gossypii* JM-310^{T}, *V. paradoxus* S110^{T}, and *E. medicae* MA11.
